# Early detection of problematic internet use among university students: psychometric validation of the PIUQ-9

**DOI:** 10.3389/fpsyg.2026.1936216

**Published:** 2026-09-30

**Authors:** Roser Granero, Fernando Fernández-Aranda, Zsolt Demetrovics, Susana Jiménez-Murcia

**Affiliations:** 1 Department of Psychobiology and Methodology, Universitat Autònoma de Barcelona - UAB, Barcelona, Spain; 2 Ciber Fisiopatología Obesidad y Nutrición (CIBERobn), Instituto Salud Carlos III, Madrid, Spain; 3 Group of Psychoneurobiology of Eating and Addictive Behaviours, Neuroscience Program, Institut d’Investigació Biomèdica de Bellvitge - IDIBELL, L’Hospitalet de Llobregat, Spain; 4 Clinical Psychology Service, Hospital Universitari de Bellvitge, L’Hospitalet de Llobregat, Spain; 5 Department of Clinical Sciences, School of Medicine and Health Sciences, Universitat de Barcelona - UB, L’Hospitalet de Llobregat, Spain; 6 College of Education, Psychology and Social Work, Flinders University Institute for Mental Health and Wellbeing, Flinders University, Bedford Park, SA, Australia; 7 Institute of Psychology, ELTE Eotvos Lorand University, Budapest, Hungary; 8 Centre of Excellence in Responsible Gaming, University of Gibraltar, Gibraltar, Gibraltar

**Keywords:** instrument validation, internet addiction disorder, online gambling disorder, online gaming disorder, PIUQ-9, problematic internet use, video games

## Abstract

**Background:**

University students extensively use the internet for academic, social, and recreational purposes, increasing the risk of developing problematic internet use (PIU). Although several instruments exist, there is a need for brief, psychometrically sound screening tools capable of identifying individuals at risk and detecting related conditions such as Internet Gaming Disorder (IGD) and Online Gambling Disorder (OGD).

**Objectives:**

This study aimed to examine the reliability and validity of the 9-item Problematic Internet Use Questionnaire (PIUQ-9) in a sample of first-year university students and to explore its screening performance for PIU and its potential utility for identifying increased risk of related conditions such as Internet Gaming Disorder (IGD) and Online Gambling Disorder (OGD).

**Methods:**

A cross-sectional study was conducted with *N* = 273 university students [180 women (65.9%), 93 men (34.1%); mean age 19.5 years, SD 1.9]. Participants were recruited from university settings and completed a self-administered battery of validated instruments assessing PIU, addictive behaviors, impulsivity, emotion regulation, and general psychopathology. Construct validity was examined using confirmatory factor analysis. Internal consistency was assessed using McDonald’s omega. Convergent validity was evaluated through Pearson correlation coefficients with related constructs. Test–retest reliability was assessed using intraclass correlation coefficients (ICC) and weighted *kappa* statistics. Receiver operating characteristic (ROC) curve analyses were conducted to determine empirically derived screening threshold for identifying high risk of PIU, IGD, and OGD.

**Results:**

Confirmatory factor analysis supported a unidimensional structure of the PIUQ-9. The scale demonstrated good internal consistency (ω = 0.82) and explained 38.5% of the total variance. Convergent validity was supported by moderate to strong correlations with related measures, including risk of internet/social network addiction (*r* = 0.666; 95%CI: 0.594–0.727), impulsivity (*r* = 0.369; 95%CI: 0.262–0.467), emotion regulation difficulties (*r* = 0.382; 95%CI: 0.276–0.479), and psychological distress (*r* = 0.312; 95%CI: 0.201–0.415) (all *P* < 0.001). Test–retest reliability was good (*ICC* = 0.753; 95%CI: 0.650–0.830), with item-level agreement ranging from moderate to substantial (weighted *kappa* = 0.435 [95%CI: 0.278–0.593] to 0.698 [95%CI: 0.593–0.803]). The area under the ROC curve was 0.824 (95% CI: 0.768–0.881). ROC analyses identified an empirically derived screening threshold of 9 for high risk of PIU, 11 for increased risk of IGD, and 13 for increased risk of OGD. These thresholds showed variable discriminatory performance, with lower specificity and positive predictive values for IGD and OGD.

**Conclusion:**

The PIUQ-9 is a brief, reliable, and valid instrument for assessing PIU among university students. This study extends previous research by providing evidence of its accuracy in identifying related behavioral addictions, such as IGD and OGD, within a population highly vulnerable to PIU yet still underrepresented in the scientific literature. The findings support its utility as an efficient screening tool in both research and applied settings, facilitating early detection and targeted prevention strategies in young adult populations.

## Graphical Abstract

**Figure:**
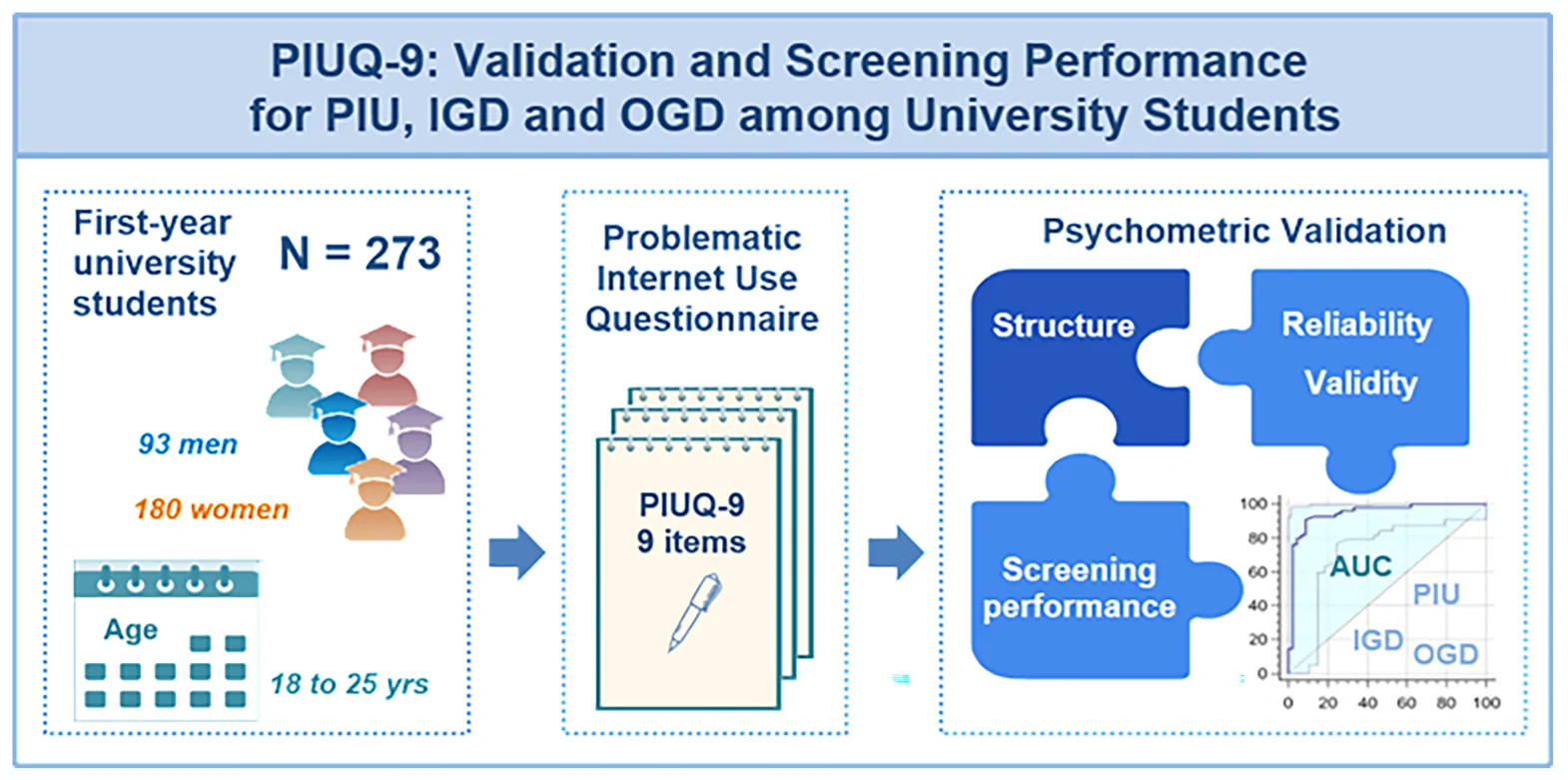

## Highlights

- The PIUQ-9 showed a robust one-factor structure and good internal consistency among young adults university students (ω = 0.82 and 38.5% explained total variance).
- Strong convergent validity was found between the PIUQ-9 with measures of online addiction risk, impulsivity, emotion dysregulation, and psychological distress.
- Excellent test-retest reliability was obtained (intraclass correlation coefficient around 0.75 for the total raw score and weighted kappa coefficients between 0.435 and 0.698 for the single items).

## 1 Introduction

Many individuals enjoy internet use without negative consequences. However, some risk factors can increase vulnerability to problematic internet use (PIU, also known as internet addiction), defined as a pattern of repeated and uncontrolled excessive online activities (such as gaming, gambling, social media use, sexual behavior, web-streaming or buying) resulting in marked functional impairment (Huang et al., 2025). PIU encompasses a multidimensional spectrum of clinical conditions, typically categorized as a behavioral addiction (Brand et al., 2025; Theopilus et al., 2025; Tóth-Király et al., 2021), being internet Gaming Disorder (IGD) and Gambling Disorder [in online modality, Online Gambling Disorder (OGD)] two fastest-growing conditions with great scientific interest during the last years (Brand et al., 2020; Király et al., 2023). The diagnostic classification of internet addiction is still controversial, and this condition is not officially recognized in the taxonomies developed by global health institutions such as the World Health Organization (ICD-11th; World Health Organization, 2022) and the American Psychiatric Association (DSM-5; American Psychiatric Association, 2013; Musetti et al., 2016). But the acknowledgment of OGD and IGD in the ICD-11 and the DSM-5 (IGD is placed in the DSM-5 into the research appendix with emerging measures and models requiring additional empirical validation) marks a shift from previous editions of these global diagnostic manuals, due to the recognition of internet addictive conditions within the psychiatric field, and encourage further empirical validation (Fineberg et al., 2018; Kaess et al., 2021; Przybylski et al., 2017).

PIU has been identified in individuals of all ages (Gjoneska et al., 2024; Kuss et al., 2014), with a weighted average prevalence of around 7% (Pan et al., 2020). Increasing incidences over time have been observed, along with mixed estimates among countries, measurement tools, and age groups. Especially elevated ranges have been observed among young ages (adolescents and young adults). The transition from adolescence to adulthood is a critical phase, marked by significant adjustments and changes that can significantly impact internet use (Anderson et al., 2017; Sánchez-Fernández and Borda-Mas, 2023). Among young people, college-university students represent a highly vulnerable subgroup. Current meta-analysis studies have estimated the global pooled prevalence of PIU in university students into the range 29% (Salpynov et al., 2024) to 42% (Liu et al., 2025), with also relevant variability across countries and income levels. The highest prevalences have been identified in low/lower-middle income countries, and an increasing trend has been reported during the past two decades which dramatically worsened during COVID-19 pandemic (Meng et al., 2022). And it is worth noting that the presence of PIU among university students is approximately twice as high as that of the general population (Zhang et al., 2018).

Different reasons contribute to the high prevalence of PIU among university students. For most of these subjects the new academic and social contexts can be perceived as highly stressful (Ferrer-Pérez et al., 2023; Karaman et al., 2019), for example coping with the new teaching methods (especially the complexity of educational programs), high expectations (both personal and familial), changes in social environments (new peers and high competitiveness), potential difficulties in covering the financial cost of university education, and challenges in balancing academic and recreational activities. Coping with these changes may lead to the emergence of multiple physical warning signs (such as sleep, eye and vision-related problems, eating, and musculoskeletal conditions), psychological symptoms (such as anxiety, depression, aggressive behavior, and social isolation) and decreased performance (probably associated with a deficit in cognitive function during the day) (Gustems-Carnicer et al., 2019; Ramón-Arbués et al., 2020; Reddy et al., 2018; Xanidis and Brignell, 2016).

In addition to the contextual stressors associated with PIU among university students, research has highlighted another crucial factor: university students use technologies for most educational purposes (such as accessing multiple learning platforms and working collaboratively), for entertainment (playing games, watching live streams, videos, or movies, or engaging in virtual reality for leisure), and for socialization (communicating and meeting new people). Consequently, excessive internet use may ultimately result in problems. The normalization of constant connectivity (reinforced by smartphones, social media platforms, and algorithm-driven content) may contribute to difficulties in self-regulation and to a blurring of boundaries between academic, social, and leisure time. Recent systematic reviews in university students have consistently shown that PIU and problematic smartphone use are emerging public health concerns associated with compulsive patterns of engagement and impaired self-regulation (Candussi et al., 2023). Continuous exposure to digital environments among university students, together with the pervasive presence of smartphones and algorithmically curated social media feeds, has been linked to increased attentional capture, reduced cognitive control, and difficulties in sustaining goal-directed behavior (Skowronek et al., 2023; Uncapher et al., 2017; Upshaw et al., 2024; Wilmer et al., 2017). These conditions have also been associated with adverse outcomes, including academic exhaustion (Chen et al., 2025), decreased academic achievement (Alzahrani and Griffiths, 2025), academic burnout (Mao et al., 2024), and poorer mental health (Chang et al., 2022). Experimental evidence further supports that temporary restriction of mobile internet access leads to improvements in sustained attention, mental health, and subjective wellbeing, suggesting that constant connectivity may directly contribute to attentional depletion and digital overload (Castelo et al., 2025). In addition, recent models of social media use highlight the role of reinforcement-based mechanisms and notification-driven engagement in promoting compulsive checking behaviors and weakening self-regulatory processes (Shannon et al., 2022).

Moreover, emerging evidence suggests that maladaptive internet use in university students may be associated with specific behavioral addictions, with marked differences in the prevalence of problematic internet use (PIU) and related endophenotypes depending on the type of online activity. In this regard, online gaming and gambling currently represent two of the fastest-growing forms of internet engagement among university students, with consistently increasing prevalence rates reported in recent studies (Akçayır et al., 2023). The expansion of online gambling platforms and microtransaction-based gaming systems has increased accessibility and lowered barriers to engagement, potentially amplifying risk in these vulnerable individuals. These dynamics may be further reinforced by peer influence and social comparison processes within digital environments, which can exacerbate feelings of inadequacy or stress. In a proportion of individuals, the university stage represents a critical period in which video gaming activity and gambling behaviors that initially begin as recreational pastimes may escalate and develop into IGD and OGD.

IGD and OGD are complex mental issues typically onset during the young age (adolescence and young adulthood), presented clinically by a persistent and recurrent need to gaming/gamble that cause significant impairment and distress. As other internet addictions, these clinical conditions are related to multiple comorbidities (Kuss and Lopez-Fernandez, 2016), dysfunctional coping strategies (Kuss et al., 2017), impulsiveness (Chamberlain et al., 2018) and difficulties in the emotion regulation strategies (Lin et al., 2023; Tessier et al., 2024). OGD have obtained pooled estimates for university students between 3.8 and 10.9% (Chiang et al., 2022), while IGD achieved around 0.4% (Grant et al., 2019) and subsyndromal symptoms into the range 8.4% (Grant et al., 2019) to 10.2% (Nowak and Aloe, 2014). And highly relevant: problematic internet gaming or gambling can exceed 60% within the subgroup of university students who gamble online regularly (Petry and Weinstock, 2007). It is therefore necessary to have reliable and valid measurement tools for rapid screening and early detection of individuals at risk, which allow intervention in the onset of problematic behaviors.

Both, IGD and OGD also share common mechanisms with PIU, including reward sensitivity, impulsivity, and emotion regulation difficulties. The interaction between psychological vulnerability, environmental stressors, and pervasive digital access highlights the need to consider internet use not only as a functional tool but also as a potential risk factor for behavioral dysregulation. This underscores the importance of early identification of at-risk students and the development of preventive interventions aimed at promoting adaptive coping strategies, digital literacy, and healthier patterns of technology use within university settings.

But the assessment of PIU is a complex task (Schlossarek et al., 2024), often considerably more difficult than substance-related addictions and other behavioral addictions, since the internet is nowadays fundamental in daily life. For many individuals, excessively problematic patterns of internet use are erroneously rationalized as necessary and socially acceptable behaviors. The numerous screening and diagnostic tests (including self-report and face-to-face measurement instruments) available for exploring PIU were designed to assess the presence of excessive/problematic use, potential associated cognitive-behavioral issues, and functional impairment (Lortie and Guitton, 2013; Mo et al., 2020). The items in these tests are typically written for assessing the specific criteria defined for related addictive behaviors in the DSM-5 and the ICD-11: internet use for a more extended period than planned, preoccupation, tolerance, withdrawal, lack of interest for alternative social/leisure activities, lack of control of internet use (unsuccessful efforts for controlling time spent), and continuous engagement with internet regarding the negative consequences. These tools are the basis for the clinical judgments about the individual’s behaviors, and for decision-making regarding the therapeutic needs. Some widely used scales are the “Problematic and Risky internet Use Screening Scale” (PRIUSS; Jelenchick et al., 2014; Moreno et al., 2016), the “Internet Addiction Test” (IAT; Moon et al., 2018), the “Generalized Problematic Internet Use Scale” (GPIUS; Caplan, 2002; Elosua et al., 2025) and the “Compulsive Internet Use Scale” (CIUS; Lopez-Fernandez et al., 2019; Meerkerk et al., 2009).

One tool that has demonstrated promising reliability and validity is the Problematic Internet Use Questionnaire (PIUQ), initially developed as a 30-item questionnaire and subsequently refined and reduced to 18 items through reliability and factor analysis studies (Demetrovics et al., 2008). This questionnaire is based on a multidimensional cognitive-behavioral model of the internet addiction (Davis, 2001), used to explain internet addiction for more than 20 years (Tunney and Rooney, 2023). This theoretical framework has proven useful in university populations, as it provides a paradigm for connecting students’ high perceived stress with the onset and progression of problematic behaviors. The model incorporates as drives the individuals’ efforts to avoid and coping from stress and its negative emotional/social states (depression, anxiety or social isolation) (Dong and Potenza, 2014); and propose a dynamic circular process that connects these motivational drivers with negative reinforces in which the individuals’ differences in the self-regulatory executive act as modulators (Zhou et al., 2023). The cognitive-behavioral model has obtained strong empirical evidence in identifying the psychological mechanisms leading to develop dysfunctional relationships with internet use, focussed on several components (Caplan, 2010; Piqueras et al., 2024): online social preference, high preoccupation with internet, escapism or mood regulation, deficient self-regulation (excessive use and loss of control), and negative outcomes (conflicts and functional impairment). Based on the cognitive-behavioral approach, the PIUQ was structured in three primary factors: (1) obsession (intrusive thoughts about internet and use of internet for coping with negative emotions); (2) neglect (functional impairment due to neglecting basic obligations such as academic/work activities, family responsibilities, or impact on sleep habits); and (3) control (loss of control over the need to go online despite negative consequences).

Two brief screening adaptations of the PIUQ have been developed, showing adequate psychometric indexes across different cultural and linguistic contexts: the 6-item version (PIUQ-6; Demetrovics et al., 2016) and the 9-item version (PIUQ-9; Laconi et al., 2018; Laconi et al., 2019). Brief screening tools can offer practical advantages in settings where large numbers of individuals need to be assessed within a limited amount of time. Because they are typically self-administered and require fewer items than comprehensive instruments, they may reduce respondent burden and facilitate their integration into large-scale screening procedures in settings such as universities, primary care, schools, workplaces, and research studies (Beidas et al., 2015; Neulinger et al., 2024). Importantly, brief instruments are not intended to replace comprehensive clinical assessments. Rather, they can serve as the first step of a multi-stage assessment process, allowing the initial identification of individuals who may require more detailed evaluation and, when appropriate, targeted intervention. The practical value of a brief instrument therefore depends not only on its reduced length, but also on whether it demonstrates adequate reliability, validity, and discriminatory capacity in the population and context in which it is intended to be used.

### 1.1 Justification and objectives

In summary, brief screening tools may be particularly useful as the first step in identifying individuals at increased risk within large and potentially vulnerable student populations. Their value in this context lies in the possibility of conducting an efficient initial assessment that can be followed, when indicated, by more comprehensive and behavior-specific evaluation. Considering the vulnerability of young university students to PIU and the increasing relevance of OGD and IGD in this population, brief screening measures supported by adequate psychometric evidence and specifically evaluated in university students may be particularly useful for large-scale research and screening procedures.

Although the PIUQ-9 has demonstrated adequate psychometric properties across multiple European countries, including Spain (Laconi et al., 2018), its validation has primarily been conducted in heterogeneous samples of general internet users. To the best of our knowledge, the instrument had not been specifically examined in a university student population. This distinction is relevant because university students, particularly those in the early stages of their university experience, may present specific patterns of internet use and may differ from general internet users in their exposure to academic, social, and recreational online activities. Therefore, psychometric properties and screening thresholds established in broader samples cannot necessarily be assumed to apply directly to this population. Moreover, PIU in university students may coexist with other increasingly prevalent online behaviors, such as problematic gaming and gambling, which warrant specific consideration. Accordingly, the objective of this study was not to demonstrate that the shorter PIUQ-9 is inherently superior to longer measures, but to examine whether this brief instrument provides adequate psychometric performance and useful screening information in young university students. We therefore aimed to establish population-specific, empirically derived screening thresholds, including thresholds associated with increased risk of IGD and OGD within this population.

Based on the theoretical conceptualization of problematic Internet use and previous psychometric research on the PIUQ, we hypothesized that the PIUQ-9 would show an adequate factorial structure, with the one-, two-, and three-factor solutions providing acceptable fit, as well as good test–retest reliability. We also expected higher PIUQ-9 scores to be positively associated with theoretically related measures of PIU, including internet-use, IGD and OGD symptoms, impulsivity, emotion regulation difficulties, and psychopathological symptoms, supporting its convergent validity.

## 2 Materials and methods

### 2.1 Participants

This manuscript presents the results of a cross-sectional study based on data from a sample of students recruited at the Autonomous University of Barcelona (Spain), after obtaining authorization from the Research Ethics Committee of the institution. Participants were recruited using a convenience sampling strategy based on voluntary participation. Inclusion criteria were being enrolled in the first year of a bachelor’s degree in Psychology and being between 18 and 25 years of age. Data collection was limited to one degree program and two academic cohorts to obtain a homogeneous sample and avoid high variability due to participants’ backgrounds, thus minimizing sampling bias.

The number of students enrolled in the first year of the Psychology program at the university was approximately 380 per academic year. The professors who were asked to collaborate were randomly selected by a member of the research team, who is also a faculty member at the university. All the professors contacted agreed to participate, allowing access to approximately 75% of the students enrolled in the 2023–2024 and 2024–2025 cohorts. Thus, approximately 570 eligible students were invited to participate in the study. The final sample included *N* = 273 participants, corresponding to an approximate response rate of 48%.

Professors collaborating in the research were asked to allow the researchers to attend one of the practical sessions to inform students about the study and invite them to participate. Two members of the research group, with extensive experience in the assessment and treatment of behavioral addictions, explained the objectives of the study and administered the assessments to the participants in the classroom. All questionnaires were reviewed immediately after completion to ensure that all items had been answered and that no questions were left unanswered before submission. Each session lasted approximately 60 min, with a group size of around 20 participants. Data were collected in May 2024 and February 2025. Participation was voluntary, and no financial or academic rewards were provided.

During the study planning phase, a minimum required sample size was calculated based on the primary aim of conducting a psychometric evaluation of the adapted version of the PIUQ-9 questionnaire. Given that the instrument comprises 9 items, the recommended sample size was determined to be between 10 and 30 participants per item.

In the total sample (*N* = 273), 180 participants were women (65.9%) and 93 were men (34.1%), with a mean age of 19.51 years (SD = 1.86). Most participants were born in Spain (256, 93.8%), and the social position index was distributed as follows: 42 (15.4%) were high, 85 (31.1%) were mean-high, 46 (16.8%) were mean, 66 (24.2%) were mean-low, and 34 (12.5%) were low.

### 2.2 Measures

#### 2.2.1 Problematic Internet Use Questionnaire-9 (PIUQ-9)

This instrument consists of nine items and can be completed in approximately 4–5 min, making it suitable for brief, self-administered screening of the presence and severity of PIU. The items are responded to in a five-point Likert scale, from never to always/almost always. This questionnaire is a brief version of the extended 18-item PIUQ (Demetrovics et al., 2008), that was originally developed for measuring internet use habits related to three specific dimensions: obsession, neglect and control difficulties. Prior studies in other countries and/or populations have reported empirical evidence regarding the psychometric properties (reliability and validity) of the PIUQ-9 for the one general factor, the second-factor structure (obsession and neglect + control), and the three-factor composition (obsession, neglect, and control difficulties). Prior validation studies for several languages (Italian, German, French, Polish, Spanish, Turkish, Hungarian, English, Chinese, Lithuanian, and Greek) have been previously published (Burkauskas et al., 2020; Koronczai et al., 2017; Laconi et al., 2019), showing good internal consistency with Cronbach’s alpha values ranging from α = 0.82 to α = 0.91 for the total scale and from 0.76 to 0.91 across its subscales. However, no psychometric data are available for young adults, specifically for university students.

In this work, the adaption to the Spanish population was based on the Internet Test Comission Guidelines for Translating and Adapting Tests (International Test Commission, 2017). The back-translation followed the next steps: (a) first, the English source was translated into the Spanish language by a member of the research team, a psychologist with large experience in behavioral addictions; (b) next, an independent psychologist next translated the target Spanish language version back into the original English language (this step was blinded to the original document); and (c) the two versions (translated and back-translated) were finally compared with the original source, to solve any potential discrepancies.

#### 2.2.2 Scale of Risk of Addiction to Social Networks and Internet (RASNI)

This is a 29 items self-report developed to assess the intensity and problematic use of social networks and internet. The questionnaire is structured in four factors, measuring the addiction severity level, social use, freaky traits (such as joining groups with specific interests, playing virtual and role-playing games, or having sexual encounters) and nomophobia (the anxiety and control distress over cell phone use). The Spanish version of the tools was used in the study (the ERA-RSI, Peris et al., 2018)), that achieved good internal consistency (Cronbach’s α = 0.90) and good temporal stability (test-retest correlations = 0.76–0.88). The internal consistency achieved in this study was α = 0.84 for the total scale, and between α = 0.73 for nomophobia to α = 0.76 for social use.

#### 2.2.3 Diagnostic Questionnaire for IGD

This is a 9 item self-report used to assess the nine criteria included in section 3 of the DSM-5 (American Psychiatric Association, 2013) for the presence of gaming disorder (Petry et al., 2014). This section includes emerging measures and models to assist the patients’ evaluation by clinicians, for conditions that need further research before they might be considered as formal disorders. The DSM-5 includes nine symptoms-criteria (preoccupations, withdrawal, tolerance, inability to reduce gaming, giving up other activities, continuing to game despite consequences, deceiving family members [or others] about the spent of time on gaming, use of gaming to cope negative moods and lost job-relationships due to the gaming), answered with a binary yes/no response scale. The cut-off required to endorse the diagnosis is 5. Previous studies reported high internal consistency for DSM-based criteria, with Cronbach’s α ranging from 0.87 to 0.98 across different samples and test-retest reliability was also acceptable (intraclass correlation coefficient between 0.71 and 0.76). The internal consistency achieved in this study was α = 0.72.

#### 2.2.4 Diagnostic Questionnaire for OGD

This is a self-report questionnaire based on the Diagnostic Questionnaire for Gaming Disorder (Stinchfield, 2003), initially developed to identify the presence of pathological gambling disorder based on the DSM criteria (diagnoses are available for the DSM-IV-TR (American Psychiatric Association, 2010) and DSM-5 versions (American Psychiatric Association, 2013)). The Spanish adaptation of this tool used in this work includes 19 items answered with a binary yes/no response scale (Jiménez-Murcia et al., 2009), and showed satisfactory convergent validity (moderate-to-high correlations with other measures of problem gambling) and high classification accuracy (hit rate = 0.95; sensitivity = 0.92; specificity = 0.99). This instrument allows for the assessment of both online and land-based gambling disorder modalities. In this study, participants were required to complete the questionnaire attending to the online gambling activity. The cut-off required to endorse the diagnosis is 4. The internal consistency achieved in this study was α = 0.75.

#### 2.2.5 Symptom Checklist-Revised (SCL-90-R)

This is a comprehensive and widely used scale including 90 items developed for assessing a large range of psychological symptoms and problematic behaviors (Derogatis, 1990). The items are responded with a 4-point Likert scale, from never to always (or almost always). The scale is structure in nine dimensions (somatization, obsessive-compulsive, interpersonal sensitivity, depression, anxiety, hostility, phobic anxiety, paranoid ideation, and psychoticism), plus three global indexes of the overall distress [Global Severity Index (GSI), Positive Symptom Distress Index (PSDI), and Positive Symptom Total (PST)]. This work used the Spanish version (Derogatis, 2002), that has shown satisfactory psychometric properties, with good internal consistency and evidence of validity across its symptom dimensions and global indices. The internal consistency calculated in the study ranged from α = 0.741 (for paranoid ideation) to α = 0.896 (for depression), and the global indexes achieved α = 0.98.

#### 2.2.6 Impulsive Behavior Scale (UPPS-P)

This is self-administered instrument containing 59 items initially designed for measuring different dimensions of the impulsive behavior (Whiteside and Lynam, 2001): lack of premeditation, lack of perseverance, sensation-seeking, negative urgency, and positive urgency. A total score is also available, calculated from the sum of all the items. The Spanish version of the UPPS-P was used in this work (Verdejo-García et al., 2010), that obtained excellent internal consistency for the total scale (Cronbach’s alpha of 0.94) and very good to excellent for the subscales (Cronbach’s alpha between 0.80 and 0.93). In our sample, internal consistency was between α = 0.867 (for sensation seeking) and α = 0.924 (for positive urgency), and the global scale achieved α = 0.95.

#### 2.2.7 Difficulties in Emotion Regulation Scale (DERS)

This self-report measures the difficulties with the emotion regulation with 36 items structured in six dimensions (Gratz and Roemer, 2004): lack of emotional awareness, lack of emotional clarity, acceptance of emotional responses, difficulties engaging in goal-directed behavior, limited access to emotion regulation strategies, and impulse control difficulties. A total score is also calculated as the sum of all the items of the scale. The Spanish version of the questionnaire was employed in the study (Hervás and Jódar, 2008), that obtained excellent internal consistency for the total scale (Cronbach’s alpha of 0.93) and very good for the subscales (Cronbach’s alpha between 0.80 and 0.87). In our work, the internal consistency was between α = 0.855 (for lack of emotional awareness) to α = 0.887 (for lack of emotional clarity), and the global scale achieved α = 0.94.

#### 2.2.8 Other sociodemographic information

This study also registered the next sociodemographic variables: participants’ sex, age, origin (born in Spain versus other countries) and the social position index (SES) (Hollingshead, 2011).

Supplementary Table 1 presents each scale and subscale used in the study, the number of items per scale, Cronbach’s alpha coefficients, and the frequency distribution for the total sample as well as separately for males and females.

### 2.3 Ethical considerations

Ethical approval was obtained from the Research Ethics Committee of the Autonomous University of Barcelona (reference code: CEEAH 6564; January 19, 2024). All participants were fully informed about the aims and procedures of the study, and written informed consent was obtained prior to participation.

Participation was voluntary, and no financial or other form of compensation was provided to participants. All data were collected and processed anonymously, ensuring confidentiality and protection of personal information. No identifiable data were recorded, and all analyses were conducted on de-identified datasets. No images, photographs, videos, screenshots, or other materials permitting the identification of individual participants are included in the manuscript or Supplementary material. Therefore, no additional consent for publication of identifiable information was required. Data were stored in secure, password-protected institutional servers with access restricted exclusively to the research team, in accordance with institutional data protection policies.

### 2.4 Statistical analysis

Statistical analysis was conducted with Stata 19 for Windows. The factor structure of the PIUQ-9 was analyses using Confirmatory Factor Analysis (CFA) implemented through structural equation modeling (SEM), and three models were tested: a one-factor model, a two-factor model, and a three-factor model. Given that the PIUQ-9 items are ordinal Likert-type items with five response categories, they were treated as approximately continuous and the models were estimated using maximum likelihood (ML). Adequate goodness-of-fit was considered for Barrett (2007): root mean square error of approximation *RMSEA* < 0.08, Bentler’s Comparative Fit Index *CFI* > 0.90, Tucker-Lewis Index *TLI* > 0.90, and standardized root mean square residual *SRMR* < 0.10. The internal consistency of the factors was assessed with Cronbach’s alpha (α) and omega (ω) values (ω coefficients were calculated because the low number of items retained in the factors) (Hayes and Coutts, 2020). The interpretation of consistency indexes was questionable for values lower than 0.70, moderate-acceptable for values higher than 0.70, good for values higher than 0.80, and excellent for values higher than 0.90.

The convergent-discriminant validity of the PIUQ-9 was assessed using Pearson’s correlation coefficient (*r*) between the total PIUQ-9 score and external measures related to PIU and overall psychopathological state. Given that the statistical significance of a correlation is strongly influenced by sample size, statistical significance was not used as the primary criterion for interpreting the magnitude of the associations. Instead, correlations were interpreted according to their effect size, using the following thresholds: poor-to-low for | *r*| < 0.24, moderate for | *r*| ≥ 0.24, good for | *r*| ≥ 0.30, and large for | *r*| ≥ 0.37 (Kelley and Preacher, 2012).

A subsample of *N* = 93 participants provided the measures of the PIUQ-9 the next 2-weeks days after the initial assessment. This information allowed testing the test-retest reliability, through the intraclass correlation coefficient (*ICC*) for the total score, and with *kappa* coefficients for each item. Given the ordinal response format of the PIUQ-9 items, unweighted and weighted *kappa* coefficients were calculated. Weighted *kappa* was estimated using both linear and quadratic weighting schemes, which assign different penalties to disagreements according to their magnitude. Linear-weighted *kappa* was prespecified as the primary weighted estimate, whereas unweighted and quadratic-weighted *kappa* was examined as a complementary analysis. For all these coefficients, the effect size was considered moderate for *ICC* > 0.50 or *kappa* > 0.40, good for *ICC* > 0.75 or *kappa* > 0.60, and large-excellent for *ICC* > 0.90 or *kappa* > 0.80 (Altman, 1991).

Receiver Operating Characteristics (ROC) analysis explored empirically derived screening thresholds of the PIUQ-9 as a screening tool for identifying the presence of PIU (as measured with the RASNI scale), OGD (as measured with the Diagnostic Questionnaire for OGD), and IGD (as measured with the Diagnostic Questionnaire for IGD). Since the optimal thresholds depends on the hypothetical prevalence of the disorders and on the costs/risks of false classifications (Zhou et al., 2002), the analysis was performed for the next criteria: a) prevalence rates for the presence of PIU, IGD and OGD equal to the study (based on the population-based sample), and b) since the main objective of screening tools in the general population is to identify at-risk individuals (which implies that costs of false negative classifications are higher than false positive ones), a sensitivity of at least 75% was required.

## 3 Results

### 3.1 Distribution of the PIUQ-9 scores

The distribution of item responses was examined as a preliminary step to assess the adequacy of the data prior to conducting the factor analyses. This procedure allows the identification of potential issues, such as null frequencies, which could affect the estimation of inter-item associations and, consequently, the factorial solution.

Table 1 contains the frequency distribution of the scores for the items of the PIUQ-9 in the sample (see also the bar chart in the upper panel of Figure 1). The items with the higher proportion of responses within the ranges often or always are 1 “feeling the need to decrease the amount of time spent online,” 2 “neglecting household chores” and 4 “incapacity to decrease the amount of time spent online.” Contrarily, the items with the higher proportion of responses in the ranges never or rarely are 7 “trying to conceal the amount of time spent online,” 8 “preoccupations in people about the time spending online” and 9 “internet use for coping with negative feelings.”

**TABLE 1:** Response distribution across PIUQ-9 items by response category (percentage %).

	Response category				
	Never (0)	Rarely (1)	Sometimes (2)	Often (3)	Always (4)
*Item 3.* Tense if cannot use long time	18.3%	38.5%	28.6%	12.1%	2.6%
*Item 6.* Tense if cannot use several days	17.9%	32.2%	27.5%	16.5%	5.9%
*Item 9.* Tense if you are not on Internet	31.5%	35.9%	22.7%	5.9%	4.0%
*Item 2.* Neglect household chores	5.9%	18.3%	44.0%	23.8%	8.1%
*Item 5.* Use Internet and not sleep	14.7%	26.4%	31.5%	20.1%	7.3%
*Item 8.* People in your life are worry	31.1%	36.6%	22.3%	7.7%	2.2%
*Item 1.* Need decrease time use	6.2%	7.0%	31.5%	39.6%	15.8%
*Item 4.* Wish decrease time use	10.6%	22.0%	37.0%	23.4%	7.0%
*Item 7*. Try to conceal time use	54.2%	23.8%	13.9%	5.5%	2.6%

The item order corresponds to the sequence of loadings in the three-factor solution presented in Table 2. Sample size: *N* = 273.

**FIGURE 1:**
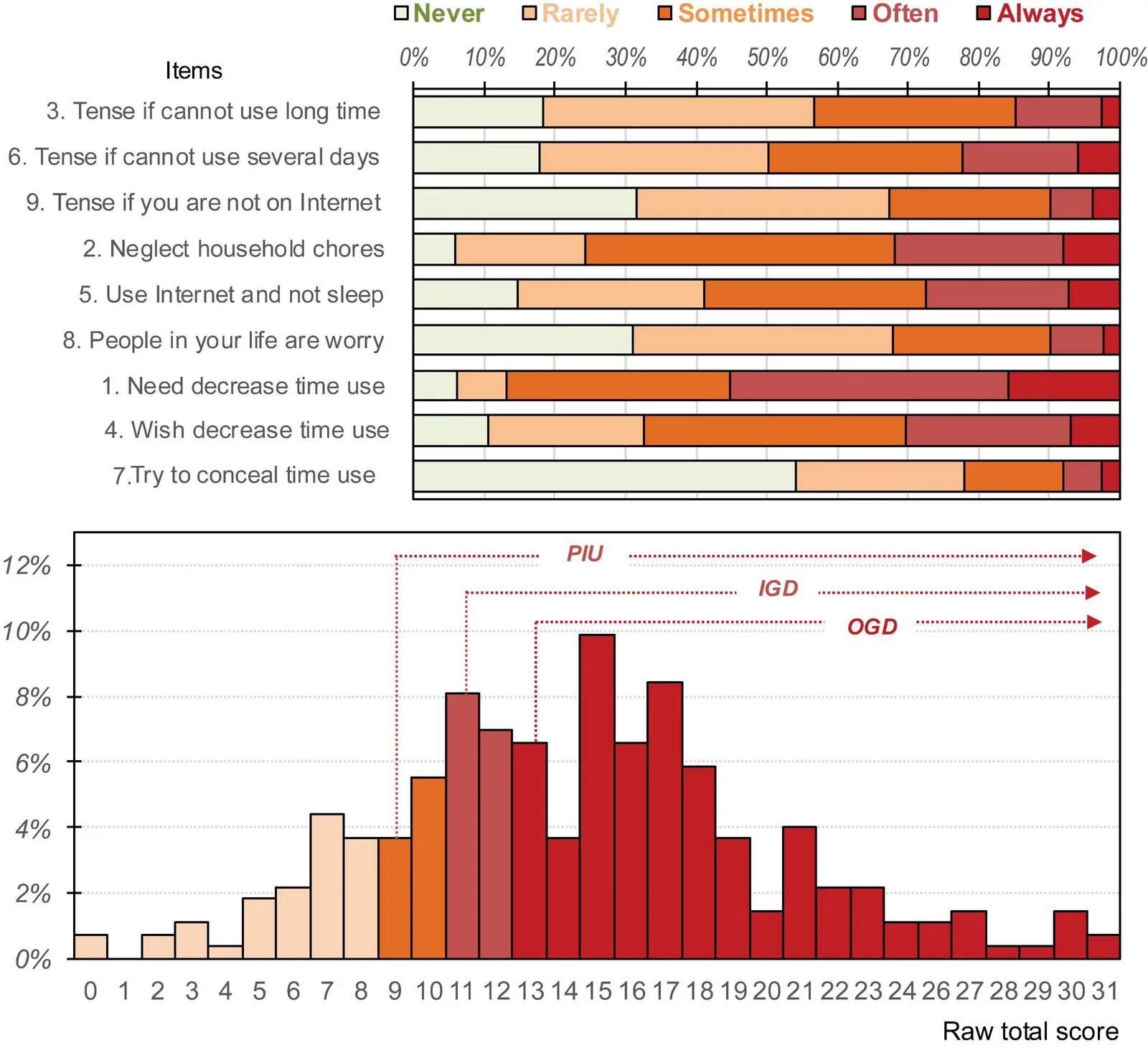
Distribution of the responses for the PIUQ-9 items and the raw total score. Upper panel: 100% stacked bar chart (percentage of individuals within each category are displayed). Lower panel: distribution of the PIUQ-9 total score; dash vertical line correspond to the suggested cut-off points for a positive screening score. PIU, problematic internet use; IGD, Internet gambling disorder; OGD, Online Gambling Disorder. Sample size: *N* = 273.

The lower panel in Figure 1 contains the graph with the distribution of the raw total score for the PIUQ-9. In the sample, scores ranged between 0 and 31, with the mean equal to 14.42 (SD = 5.82).

### 3.2 Assessment of the factor structure of the PIUQ-9

Figure 2 contains the standardized factor loadings obtained in the CFA for the three models tested in the study for the PIUQ-9 (one factor, two factors, and three factors). Additional results are displayed in Table 2. All the solutions achieved goodness-of-fit (fit statistics into the adequate range), being the best adjustment for the one-factor structure [RMSEA = 0.067 (*P* = 0.186); CFI = 0.982; TLI = 0.946; and SRMR = 0.045] and the poorest for the three-factor structure [RMSEA = 0.076 (*P* = 0.058); CFI = 0.967; TLI = 0.930; and SRMR = 0.059]. For all the models, statistically significant standardized factor loadings above 0.30 were obtained for all items. In the one-factor solution, these loadings were generally moderate in magnitude, whereas higher loadings were observed in the multidimensional solutions. The internal consistency (as assessed by the coefficients) was also good for the one general factor (ω = 0.82 for the scale) and the bi-factor (ω = 0.92 for obsession and ω = 0.82 for neglect/control), but poorer for the three-factor (ω = 0.92 for obsession, ω = 0.73 for neglect, and ω = 0.72 for control). The percent of explained variance ranged between 38.5% for the one-factor solution and 68.9% for the three-factor solution. For the two-factor solution, the correlation between obsession with neglect/control was high (*r* = 0.401). For the three-factor solution, the correlation was high between neglect with obsession (*r* = 0.524) and neglect with control (*r* = 0.517), and poor between obsession with control (*r* = 0.190).

**FIGURE 2:**
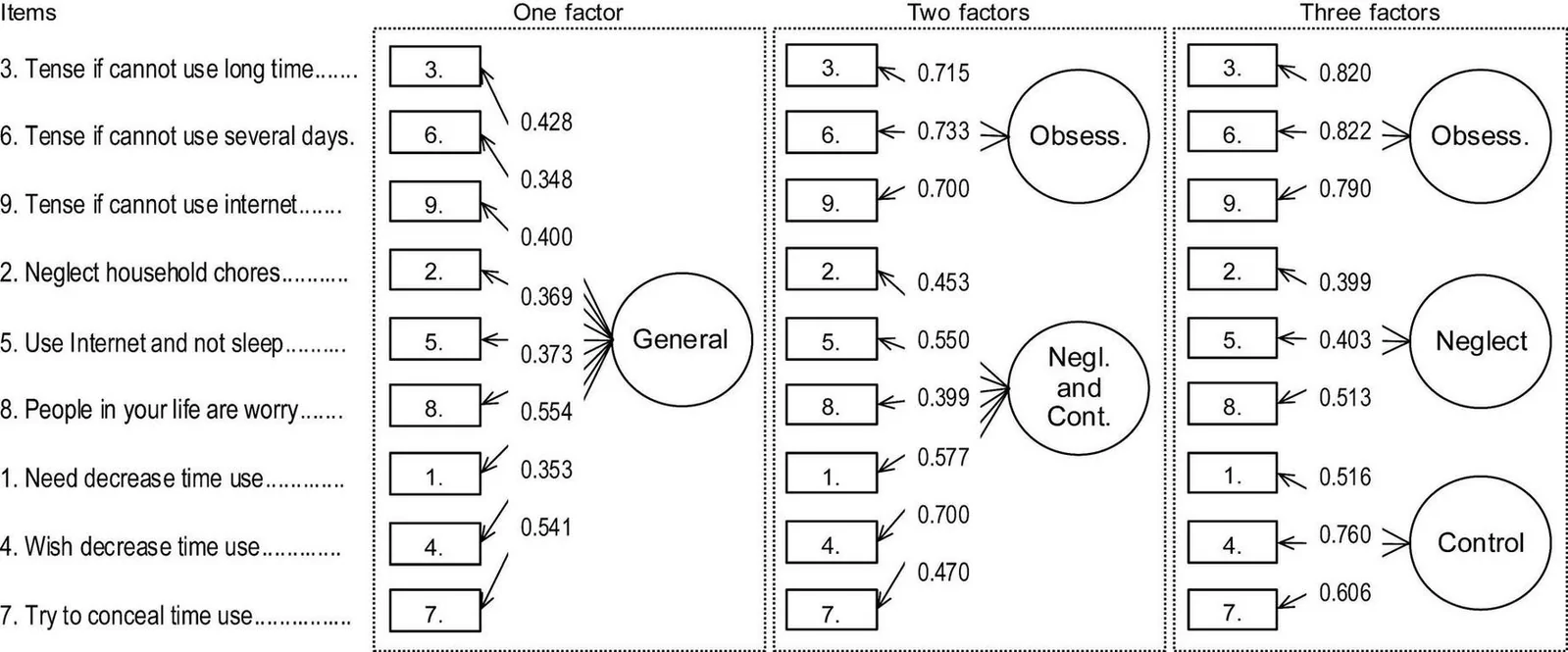
Path diagrams of the SEM: standardized factor loadings. The item order corresponds to the sequence of loadings in the three-factor solution. Sample size: *N* = 273.

**TABLE 2:** Results obtained in the confirmatory factor analysis of the PIUQ-9.

Solution	One factor	Two factors		Three factors		
	General	Obsession	Neg/Cont	Obsession	Neglect	Control
Dimensionality Cronbach’s alpha (α)	0.794	0.852	0.730	0.852	0.710	0.708
Omega (ω)	0.820	0.915	0.818	0.915	0.725	0.717
Explained variance	38.48	58.21		68.86		
Fit statistics RMSEA	0.067	0.075		0.076		
RMSEA *P-close*	0.186	0.105		0.058		
CFI	0.982	0.972		0.967		
TLI	0.946	0.932		0,930		
SRMR	0.045	0.055		0.059		

The item order corresponds to the sequence of loadings in the three-factor solution. RMSEA, Root Mean Square Error of Approximation. CFI, Comparative Fit Index. TLI, Tucker-Lewis Index. SRMR, standardized root mean residual. Sample size: *N* = 273.

Based on the global fit indices derived from the SEM (RMSEA, TLI, CFI and SRMR), the one-factor solution showed the most favorable overall fit and was therefore retained as the primary model for interpretation, followed by the two-factor model. It should be noted that the standardized factor loadings in the two-factor solution were higher than those observed in the unidimensional model, indicating a better representation of the item–factor relationships when the scale is specified with two correlated factors. This suggests that, although the one-factor solution is a plausible and acceptable model (with all factor loadings being statistically significant and above 0.30, and showing slightly better global fit indices), the two-factor solution cannot be ruled out, as it also provides an adequate representation of the data and yields stronger item–factor associations. Furthermore, the moderate factor loadings observed in the unidimensional solution suggest that the common latent factor does not account for a large proportion of item variance, supporting the possibility that problematic internet use may contain distinguishable subdimensions.

Regarding the three-factor model, although it achieved acceptable fit indices, the lower reliability estimates (omega values) for the neglect and control dimensions and the more complex structure provided less support for its adoption as the preferred representation of the data.

Supplementary Table 2 contains the normative data (percentile and T-standardized scores) for the three solutions tested in the study.

### 3.3 Assessment of the test-retest reliability

Table 3 contains the test-retest reliability indexes. For each item, the linear weighted *kappa* coefficients were in the good range (between *kappa* = 0.435 for the item 2 “neglect household chores” to *kappa* = 0.698 for item 9 “internet use for coping with negative feelings”). For the factor scores, the test-retest reliability was good for the one general factor (*ICC =* 0.753) and the bi-factor model (*ICC* = 0.798 for obsession and *ICC* = 0.761 for neglect + control). For the three-factor solution, the test-retest reliability was good for obsession (*ICC* = 0.798) and control (*ICC* = 0.771) and moderate for neglect (*ICC* = 0.670)

**TABLE 3:** Test-retest reliability of the PIUQ-9: kappa and intraclass correlation coefficients.

	Unweighted coefficient			Weighted coefficient: linear			Weighted coefficient: quadratic		
Items	Kappa	*P*-value	95% CI	Kappa	*P*-value	95% CI	Kappa	*P*-value	95% CI
Item 3	0.468	< 0.001	0.323; 0.612	0.593	< 0.001	0.465; 0.721	0.703	< 0.001	0.563; 0.844
Item 6	0.534	< 0.001	0.394; 0.674	0.614	< 0.001	0.484; 0.745	0.700	< 0.001	0.572; 0.829
Item 9	0.560	< 0.001	0.469; 0.723	0.698	< 0.001	0.593; 0.803	0.782	< 0.001	0.660; 0.903
Item 2	0.247	< 0.001	0.077; 0.416	0.435	< 0.001	0.278; 0.593	0.629	< 0.001	0.485; 0.772
Item 5	0.330	< 0.001	0.183; 0.476	0.474	< 0.001	0.343; 0.606	0.620	< 0.001	0.491; 0.749
Item 8	0.563	< 0.001	0.438; 0.688	0.650	< 0.001	0.539; 0.761	0.724	< 0.001	0.594; 0.854
Item 1	0.345	< 0.001	0.187; 0.503	0.545	< 0.001	0.409; 0.681	0.718	< 0.001	0.604; 0.833
Item 4	0.508	< 0.001	0.365; 0.650	0.598	< 0.001	0.463; 0.733	0.692	< 0.001	0.560; 0.825
Item 7	0.514	< 0.001	0.380; 0.649	0.598	< 0.001	0.476; 0.720	0.660	< 0.001	0.505; 0.816
	**1-Factor**		**2-Factors**			**3-Factors**			
Factor scores	General		Obsession	Neglect / Control		Obsession	Neglect	Control	
ICC	0.753		0.798	0.761		0.798	0.670	0.771	
*P*-value	< 0.001		<0.001	< 0.001		<0.001	< 0.001	<0.001	
95%CI	0.650, 0.830		0.710, 0.861	0.654, 8.837		0.710, 0.861	0.541, 0.768	0.652, 0.859	

Item 3: tense if cannot use long time. Item 6: tense if cannot use several days. Item 9: tense if you are not on Internet. Item 2: neglect household chores. Item 5: spend time when you’d rather sleep. Item 8: people in your life are worry. Item 1: should decrease (amount of time). Item 4: wish decrease (amount of time). Item 7: try to conceal (amount of time). ICC, intraclass correlation coefficient; 95%CI, 95% confidence interval. Weighted *kappa* coefficients were calculated using linear and quadratic weighting schemes. Linear-weighted *kappa* was considered the primary weighted estimate. Subsample size: *n* = 93.

### 3.4 Assessment of the convergent discriminative validity

All external measures included in the correlational analyses were theoretically related to problematic Internet use; therefore, positive associations were expected and the results are reported as evidence of convergent validity rather than discriminant validity.

Table 4 contains the correlation matrix with the associations between the PIUQ-9 factor scores with the external measures of the study (correlations showing moderate-to-large effect sizes are presented in bold, as indicated in the table note). The one general factor obtained relevant positive correlations with all the measures, except for the RASNI freaky traits, the number of DSM-5 criteria for OGD, the UPPS-P sensation seeking, the DERS lack of emotional awareness and the SCL-90R somatization and phobic anxiety.

**TABLE 4:** Correlation coefficients between the PIUQ-9 factor scores and the external measures.

	General			Obsess			Neglect / Control			Neglect			Control		
	*r*	95%CI		*r*	95%CI		*r*	95%CI		*r*	95%CI		*r*	95%CI	
RASNI addiction symptoms	**0.754** ^†^	0.698	0.801	**0.629** ^†^	0.551	0.696	**0.640** ^†^	0.564	0.705	**0.683** ^†^	0.614	0.742	**0.442** ^†^	0.341	0.533
RASNI social use	**0.309** ^†^	0.198	0.413	0.198	0.081	0.309	**0.303** ^†^	0.191	0.407	**0.253** ^†^	0.138	0.361	**0.272** ^†^	0.158	0.378
RASNI freaky traits	0.162	0.044	0.275	0.202	0.085	0.313	0.091	-0.028	0.207	0.169	0.051	0.282	-0.004	-0.123	0.115
RASNI nomophobia	**0.526** ^†^	0.434	0.607	**0.621** ^†^	0.542	0.689	**0.320** ^†^	0.209	0.423	**0.428** ^†^	0.326	0.520	0.140	0.022	0.254
RASNI total	**0.666** ^†^	0.594	0.727	**0.611** ^†^	0.531	0.680	**0.526** ^†^	0.434	0.607	**0.583** ^†^	0.499	0.656	**0.342** ^†^	0.233	0.443
Gaming disorder: number criteria	**0.266** ^†^	0.152	0.373	**0.359** ^†^	0.251	0.458	0.131	0.012	0.246	**0.273**	0.159	0.379	-0.032	-0.150	0.087
Gambling disorder: number criteria	0.106	-0.013	0.222	0.061	-0.058	0.178	0.109	-0.010	0.225	0.176	0.059	0.289	0.020	-0.099	0.138
UPPS-P lack premeditation	**0.252** ^†^	0.137	0.360	**0.428** ^†^	0.326	0.520	0.067	-0.052	0.184	**0.254**	0.139	0.362	-0.117	-0.232	0.002
UPPS-P lack perseveration	**0.362** ^†^	0.254	0.461	**0.478** ^†^	0.381	0.565	0.188	0.071	0.300	**0.322**	0.211	0.424	0.022	-0.097	0.140
UPPS-P sensation seeking	0.098	-0.021	0.214	0.015	-0.104	0.133	0.129	0.010	0.244	0.174	0.056	0.287	0.055	-0.064	0.173
UPPS-P positive urgency	**0.324** ^†^	0.213	0.426	**0.408** ^†^	0.304	0.502	0.182	0.065	0.294	**0.324**	0.213	0.426	0.010	-0.109	0.129
UPPS-P negative urgency	**0.331** ^†^	0.221	0.433	**0.449** ^†^	0.349	0.539	0.162	0.044	0.275	**0.287**	0.174	0.392	0.007	-0.112	0.126
UPPS-P total	**0.369** ^†^	0.262	0.467	**0.475** ^†^	0.378	0.562	0.200	0.083	0.311	**0.370**	0.263	0.468	-0.003	-0.122	0.116
DERS non-acceptance emotions	**0.278** ^†^	0.165	0.384	**0.246** ^†^	0.131	0.354	0.224	0.108	0.334	0.148	0.030	0.262	**0.236** ^†^	0.121	0.345
DERS diff. directed behaviors	**0.322** ^†^	0.211	0.424	**0.377** ^†^	0.270	0.474	0.200	0.083	0.311	**0.246**	0.131	0.354	0.111	-0.008	0.227
DERS impulse control difficulties	**0.291** ^†^	0.178	0.396	**0.425** ^†^	0.323	0.518	0.123	0.004	0.238	0.218	0.102	0.328	0.006	-0.113	0.125
DERS lack emotional awareness	0.141	0.023	0.255	**0.240** ^†^	0.125	0.349	0.036	-0.083	0.154	0.071	-0.048	0.188	-0.003	-0.122	0.116
DERS limited access emotions	**0.329** ^†^	0.219	0.431	**0.392** ^†^	0.287	0.488	0.197	0.080	0.309	**0.247**	0.132	0.355	0.102	-0.017	0.218
DERS lack emotional clarity	**0.257** ^†^	0.143	0.365	0.226	0.110	0.336	0.210	0.094	0.321	0.119	0.000	0.234	**0.240** ^†^	0.125	0.349
DERS total	**0.382** ^†^	0.276	0.479	**0.452** ^†^	0.352	0.542	0.233	0.118	0.342	**0.251**	0.136	0.359	0.158	0.040	0.272
SCL-90R somatization	0.179	0.062	0.292	0.108	-0.011	0.224	0.180	0.063	0.292	0.122	0.003	0.237	0.187	0.070	0.299
SCL-90R obsessive/compulsive	**0.302** ^†^	0.190	0.406	0.072	-0.047	0.189	**0.381** ^†^	0.275	0.478	0.192	0.075	0.304	**0.461** ^†^	0.362	0.550
SCL-90R interpersonal sensitivity	**0.286** ^†^	0.173	0.391	0.141	0.023	0.255	**0.310** ^†^	0.199	0.414	0.202	0.085	0.313	**0.330** ^†^	0.220	0.432
SCL-90R depressive	**0.237** ^†^	0.122	0.346	0.029	-0.090	0.147	**0.316** ^†^	0.205	0.419	0.177	0.060	0.290	**0.362** ^†^	0.254	0.461
SCL-90R anxiety	**0.257** ^†^	0.143	0.365	0.147	0.029	0.261	**0.264** ^†^	0.150	0.371	0.167	0.049	0.280	**0.287** ^†^	0.174	0.392
SCL-90R hostility	**0.297** ^†^	0.185	0.402	**0.334** ^†^	0.224	0.435	0.193	0.076	0.305	**0.288** ^†^	0.175	0.393	0.057	-0.062	0.175
SCL-90R Phobic anxiety	0.227	0.111	0.337	0.139	0.021	0.254	0.226	0.110	0.336	0.100	-0.019	0.216	**0.283** ^†^	0.170	0.389
SCL-90R paranoid Ideation	**0.319** ^†^	0.208	0.422	0.221	0.105	0.331	**0.302** ^†^	0.190	0.406	**0.247** ^†^	0.132	0.355	**0.276** ^†^	0.163	0.382
SCL-90R psychotic	**0.275** ^†^	0.162	0.381	0.127	0.008	0.242	**0.303** ^†^	0.191	0.407	0.161	0.043	0.274	**0.357** ^†^	0.249	0.456
SCL-90R GSI	**0.312** ^†^	0.201	0.415	0.150	0.032	0.264	**0.340** ^†^	0.231	0.441	0.215	0.099	0.325	**0.369** ^†^	0.262	0.467
SCL-90R PST	**0.275** ^†^	0.162	0.381	0.112	-0.007	0.228	**0.313** ^†^	0.202	0.416	0.164	0.046	0.277	**0.370** ^†^	0.263	0.468
SCL-90R PSDI	**0.295** ^†^	0.183	0.400	0.174	0.056	0.287	**0.300** ^†^	0.188	0.404	**0.254** ^†^	0.139	0.362	**0.269^†^**	0.155	0.376

† Bold: effect size within the ranges mild/moderate to high/large (| *r*| > 24). 95%CI, 95% confidence interval. Sample size: *N* = 273. RASNI, Scale of Risk of Addiction to Social Networks and Internet (ERA-RSI in Spanish version); UPPS-P, Impulsive Behavior Scale; DERS, Difficulties in Emotion Regulation Scale; SCL-90R, Symptom Checklist-90-Revised.

For the bi-factor solution of the PIUQ-9, the dimension measuring the obsession behavior was related to the RASNI scores (except for social use and freaky traits), the number of criteria for IGD, impulsivity (except for sensation seeking), difficulties with emotional regulation (except for the lack of emotional clarity), and the SCL-90R hostility score. Regarding the neglect + control dimension, positive associations were achieved with the RASNI scores (except for freaky traits), and the psychopathology state as measured by the SCL-90R (except for somatization, hostility and phobic anxiety).

Considering the three-factor solution of the PIUQ-9, the factor measuring neglect behavior correlated with the RASNI scores (except for freaky traits), the number of DSM-5 criteria for IGD, the impulsivity (except sensation seeking), the emotion dysregulation (concretely in the directed behaviors, limited access to emotions and total), and the psychopathology level for hostility, paranoid ideation and PSDI. And the PIUQ-9 factor measuring the control behavior was associated with the RASNI (concretely with the addiction level, social use and total scores), the difficulties in the emotion regulation (non-acceptance of emotions and lack of emotional clarity), and all the scores in the SCL-90R (except somatization and hostility).

### 3.5 Selection of the empirically derived screening threshold

The number of participants with positive score in the RASNI was n = 70 (prevalence equal to 25.6%). ROC analysis selected 9 as the empirically derived screening threshold in the PIUQ-9 for identifying positive score in the RASNI (see Figure 3 and Table 5). This score obtained sensitivity = 98.6% and specificity = 80.3%. For prevalences of RASNI positive scores equal to the study, the predictive value for a negative screening score was 99.4%, and for a positive screening score 63.3%. The area under the ROC curve was 0.824 (95% confidence interval: 0.768–0.881).

**FIGURE 3:**
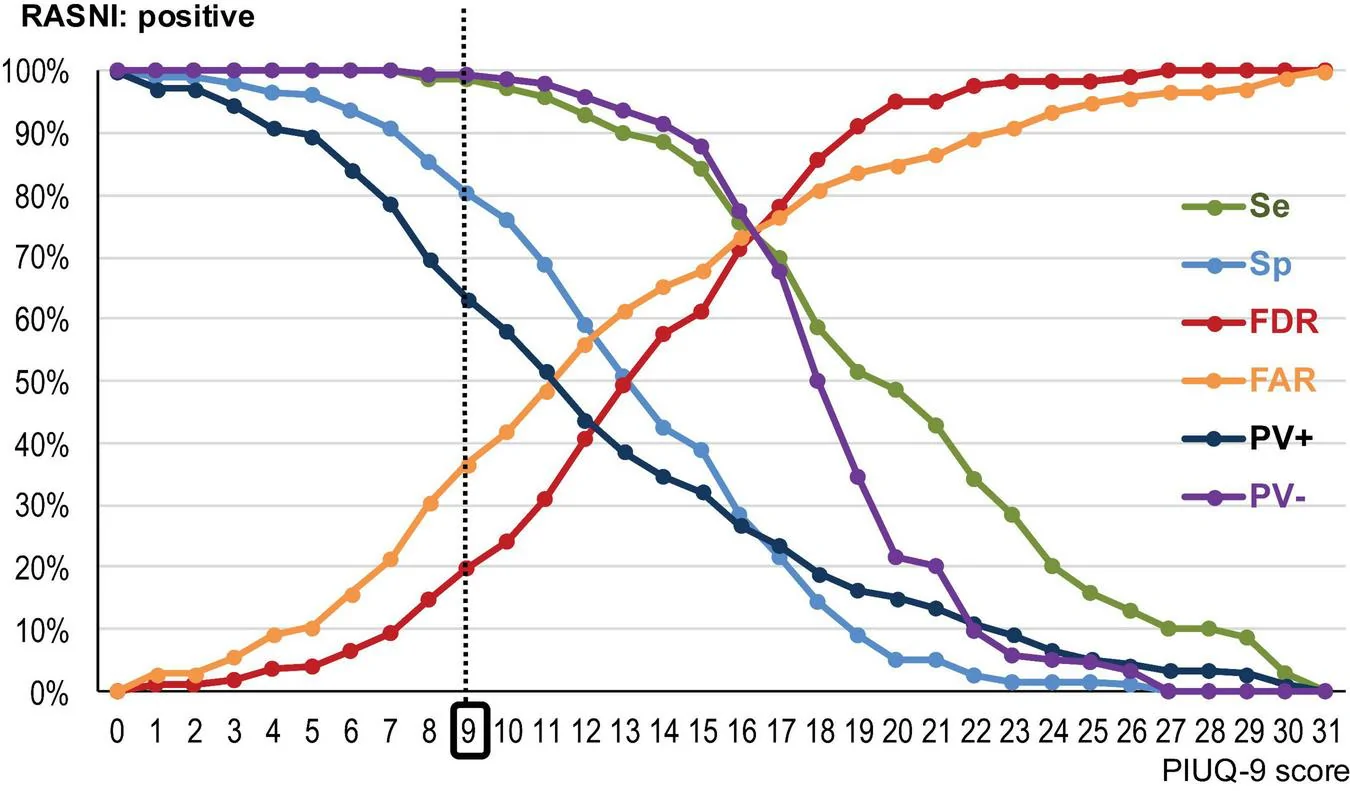
Selection of the optimal cut-off for the PIUQ-9 one general factor to identify Internet addiction (based on the RASNI): ROC analysis. Se, sensitivity (%); Sp, specificity (%); FDR, false discovery rate (%); FAR, false alarm rate (%); PPV, predictive value for a positive screening score (%); PNV, predictive value for a negative screening score (%); RASNI, Scale of Risk of Addiction to Social Networks and Internet (ERA-RSI in Spanish version). The dashed vertical line indicates the optimal cutoff point. Sample size: *N* = 273.

**TABLE 5:** Diagnostic accuracy metrics for optimal cutoff points of the PIUQ-9.

Criterion	Cut	Se	Sp	FDR	FAR	PPV	PNV	AUC	95%CI AUC	
RASNI: high risk	9	98.6%	80.3%	19.7%	36.7%	63.3%	99.4%	0.824	0.768	0.881
Gaming or gambling	11	73.0%	35.3%	64.7%	60.5%	39.5%	69.3%	0.663	0.601	0.731
Gaming	11	74.5%	35.8%	64.2%	62.2%	37.8%	72.7%	0.661	0.590	0.732
Gambling	13	76.0%	48.0%	52.0%	86.0%	14.0%	94.7%	0.685	0.587	0.803

Se, sensitivity (%); Sp, specificity (%); FDR, false discovery rate (%); FAR, false alarm rate (%). PPV, predictive value for a positive screening score (%); PNV, predictive value for a negative screening score (%); AUC, area under the ROC curve; 95%CI, 95% confidence interval; RASNI, Scale of Risk of Addiction to Social Networks and Internet (ERA-RSI in Spanish version). Sample size: *N* = 273.

In addition to identifying the screening threshold for PIU as measured by the RASNI, we explored whether PIUQ-9 scores could discriminate between participants with and without elevated risk of problematic gaming or gambling, as defined by established reference measures. Given the conceptual overlap between problematic internet use and specific online behaviors such as gaming and gambling, we examined whether different PIUQ-9 score ranges were associated with increased risk profiles for these behaviors. These analyses were exploratory and were intended to assess the potential screening value of the PIUQ-9, rather than to establish clinically validated diagnostic thresholds for IGD or OGD.

Regarding the accuracy of the PIUQ-9 for identifying online gambling and gaming, the number of participants with 1–4 DSM-5 criteria for IGD was *n* = 89 (prevalence for problematic internet gaming equal to 32.60%), and *n* = 5 individuals (prevalence equal to 1.83) met the threshold for IGD (see Figure 4). Regarding OGD, *n* = 19 participants reported between 1 and 3 DSM-5 criteria (prevalence for problematic pathological gambling equal to 6.96%), while *n* = 6 subjects (2.20%) met the criteria for OGD. In addition, the presence of problematic internet gaming *or* online gambling was identified in *n* = 90 individuals (32.97%), and *n* = 10 (3.66%) presented the diagnosis of OGD *or* IGD.

**FIGURE 4:**
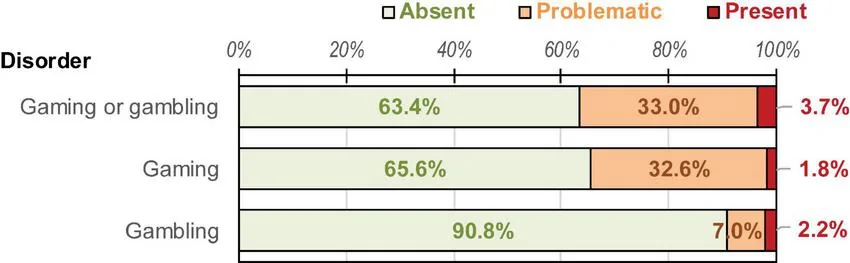
Presence of the gaming and gambling disorder in the study: prevalence estimates. Sample size: *N* = 273.

For the ROC analyses, 3 reference classifications were considered: (a) problematic or disordered gaming or gambling, identified in 90 participants; (b) problematic or disordered gaming, identified in 89 participants; and (c) problematic or disordered gambling, identified in 19 participants. These classifications included participants reporting subthreshold DSM-5 symptoms as well as those meeting the full diagnostic criteria. ROC procedures identified as 11 the screening threshold for identifying the presence of problematic or disordered internet gaming, 13 for identifying the presence of problematic or disordered online gambling was, and 11 for identifying the presence of problematic or disordered IGD or OGD (see Table 5 and Supplementary Figure 1). These thresholds, obtained for prevalence values equal to the study, achieved sensitivity higher than 0.70, but low specificity and predictive positive values (PV+). And consequently, the risk of false alarm rate (FAR, the complement of the specificity) and false discovery rate [FDR, the complement of the positive predictive value (PV+)], were also high.

## 4 Discussion

The current study assessed the reliability and validity of the Spanish version of the PIUQ-9 in a sample of university students. The results obtained in the CFA supported the adequacy of the one-factor, two-factor, and three-factor solutions, with the one-factor model showing the most favorable global fit indices. The PIUQ-9 has evidenced test-retest reliability (suggesting the temporal consistence of the items for measuring the same underlying construct), and convergent validity with external constructs related to the PIU (such as scales of risk addiction to social networks and internet, impulsivity, emotion regulation and psychopathology symptoms). These results showed that this brief screening tool has good properties for identifying individuals with excessive internet use, and who are at high risk of OGD and IGD.

Regarding the factor structure of the PIUQ-9, the CFA results showed that the unidimensional, two-factor, and three-factor solutions all provided adequate to acceptable fit to the data. Thus, the findings do not indicate that one factor structure should be considered unequivocally superior to the others. These results support the use of the PIUQ-9 as either a unidimensional measure of overall PIU or as a multidimensional measure through its specific subscales. Accordingly, the most appropriate scoring approach may depend on the specific objectives and research questions of each study, with the total score being appropriate when a global assessment is intended and the subscales being useful when the different dimensions of PIU are of specific interest.

The PIUQ-9 obtained convergent validation with the RASNI, particularly with the addiction symptoms and total scales. Overall, the highest correlation coefficients were achieved between the RASNI and the general factor. One exception was the RASNI freaky traits, that obtained null/low relationships with the PIUQ-9. In the field of mental health, freaky traits scales usually assess specific interests (e.g., new technologies or video games), a preference for unconventional activities, or certain cognitive and personality styles (such as introversion) (Peris et al., 2018). In the case of the RASNI questionnaire, the freaky traits factor includes items for evaluating the following behaviors: spending time on the internet to join interest-based groups, participating in virtual and/or role-playing games, seeking sexual information, sending erotic messages, visiting erotic websites, and engaging in sexual encounters. These items reflect specific interests, but do not necessarily indicate problematic, uncontrolled, or dysfunctional internet use. Previous studies have shown that individuals with high scores on freaky traits may engage in intensive use of online social media and/or other virtual platforms without such behaviors interfering with their academic, professional, and social functioning (Fauth-Bühler and Mann, 2017; Kardefelt-Winther, 2014). Therefore, high scores on freaky traits may reflect intense exploratory activity in specific areas or high engagement with like-minded communities (Gocłowska et al., 2019; Oksanen et al., 2024). In contrast, high scores on PIU are commonly associated with avoidant or escapist motivations (e.g., to cope and avoid negative mood states, anxiety and depression) and involve symptoms such as tolerance, withdrawal, loss of control, and functional impairment (McCain et al., 2015; Peeples et al., 2018). Regarding the relationship between elevated freaky traits and neuroticism, it is also possible that individuals with low self-esteem or social skills difficulties may prefer online activities because they involve less anxiety by reducing face-to-face communication demands (Wang, 2017; Weinstein et al., 2015). Again, this pattern would represent intensive and specialized internet use, not necessarily problematic/addictive behavior.

Positive correlations were also observed between the number of criteria for IGD with the PIUQ-9, one general factor, obsessions and neglect. However, only poor correlations were obtained between the number of criteria for OGD and the PIUQ-9. These results are consistent with the distribution of the OGD and IGD severity levels registered in the study: 94 students (34.4%) met the criteria (DSM-5 threshold) for problematic or disordered online gaming, but only 25 students (9.2%) met criteria for problematic or disordered online gambling. The small number of participants with problematic or disordered internet gambling leads to a severity measure (the number of DSM-5 criteria in our work) based on a limited subset of individuals, and therefore to a very low variability for the measurement score. In other words, because few students reported internet gambling-related problems, the range of severity levels was restricted, reducing score variability and potentially attenuating correlations with the PIUQ-9 while decreasing the statistical power to detect true associations (Bland and Altman, 2011; Miciak et al., 2016).

Notably, the PIUQ-9 has one general factor that obtained relevant correlations with impulsivity (except for sensation seeking), the difficulties in emotion regulation (except for lack of emotional awareness) and the psychopathology levels (except for somatization and phobic anxiety). This correlation pattern suggests that PIU among university students can be driven as a mechanism for coping with negative psychological states (such as depression, anxiety and global distress) modulated by high levels of impulsivity plus deficits in the emotion regulation processes (Gioia et al., 2021; Li et al., 2021). Recent studies have also documented negative urgency (the predisposition to act impulsively when faced with negative emotions) as a key facet of impulsivity that helps explain the association between impulsivity and negative emotionality (Quintero et al., 2020; Willie et al., 2022). Individuals with high levels negative urgency are characterized by prone to engage in maladaptive avoidance behaviors (such as PIU) as immediate escape mechanisms from negative affect (Chester et al., 2017; Zorrilla and Koob, 2019). This pattern aligns with a compulsive pathway, wherein repetitive engagement is maintained not for intrinsic reward but for the alleviation of emotional distress, consistent with mechanisms of negative reinforcement (Liu et al., 2024). Neurocognitive and neuroimaging evidence further supports the involvement of impulsivity and emotion dysregulation in PIU and gambling, showing alterations in emotional processing, response inhibition, executive functioning, and reward-related mechanisms (Li et al., 2020; Navas et al., 2017; Park et al., 2017; Shin et al., 2021; Wojtczak et al., 2024). These findings are consistent with the role of negative reinforcement in the persistence and escalation of problematic behaviors (Baker et al., 2004; Brand et al., 2016). Therefore, early assessment of impulsivity, emotion regulation difficulties, and comorbid psychopathology may contribute to the identification of individuals at risk of PIU and facilitate timely intervention (Brand et al., 2019; Fineberg et al., 2018; Quaglieri et al., 2021). In clinical settings, assessing these transdiagnostic dimensions may also contribute to more precise treatment planning and prevention of comorbidities (Brand et al., 2019; Fineberg et al., 2018; González-Roz et al., 2024; Trautmann et al., 2025; Vassileva and Conrod, 2019).

The PIUQ-9 obtained good validity for identifying individuals at high risk of PIU (as measured by the RASNI). The area under the ROC curve was very good, as well as the sensitivity and specificity coefficients the threshold 9 points. Regarding the use of the PIUQ-9 as a brief screening scale for identifying OGD and IGD, it should be kept in mind that the use of these instruments in general population or highly vulnerable sub-populations is primary aimed to identify individuals at risk for mental health states, and who need further exhaustive assessments to confirm/discard the presence of disorders (American Psychological Association, 2020; Health Knowledge, 2023). Screening instruments are not diagnostic procedures but improve early detection of problematic behaviors, and therefore high sensitivity is a key requirement. Positive scores for screening scales are not indication of the presence of the disorder, but to the need of additional precise assessment for deciding the need of treatment. Regarding this point, PIU is a nonspecific construct that encompasses a range of maladaptive online behaviors, and studies have distinguished between the *generalized* and *specific* forms of internet addiction (Davis, 2001): the generalized type involves a marked engagement with the internet without a clearly defined purpose, whereas the specific type applies to excessive use focused on concrete online activities (such as gaming or gambling, but also social media, or pornography consumption). This distinction between general versus specific has generated a scientific growing interest for examining the distinct symptoms-behaviors related with PIU with the aim of conceptualizing this behavioral addiction as a spectrum of internet-mediated activities that share both common and unique features (Lopez-Fernandez, 2018; Sánchez-Fernández et al., 2024; Shi et al., 2025; Starcevic and Aboujaoude, 2017). Within this framework, the PIUQ-9 is a questionnaire designed to assess generalized PIU, and accordingly, a positive screening score should be considered a warning sign for potentially maladaptive diverse online activities, requiring a targeted follow-up assessment to determine the specific activity or activities responsible for the problems. And since the presence of this complex condition among young individuals is associated with many comorbid psychological symptoms (such as depression, anxiety, and eating or sleep disturbances, and other comorbid disorders) (Cai et al., 2023; Hidalgo-Fuentes et al., 2023), but also with academic failure (Guo et al., 2021; Wei et al., 2025), identifying individuals with a PIUQ-9 positive screening should involve a comprehensive evaluation of their bio-psycho-social functioning aimed at obtaining a specific profile to guide an appropriate and targeted intervention.

The previous consideration is particularly important when interpreting the PIUQ-9 screening thresholds in relation to OGD and IGD. The PIUQ-9 was not specifically designed to detect these distinct forms of online addictive behaviors; therefore, it should not be interpreted as a diagnostic instrument for gaming or gambling disorders. Rather, its potential value lies in its use as a first-step screening tool. In this context, the observed sensitivity values may support the potential utility of the PIUQ-9 for identifying individuals who may be at increased risk and who could benefit from more specific follow-up assessment using dedicated instruments. However, these values should be interpreted in conjunction with the corresponding specificity estimates and within the limitations of the self-report reference measures used in the present study. In particular, screening tools applied in populations with relatively low prevalence rates may generate a substantial number of false-positive classifications, potentially resulting in unnecessary additional assessments or inappropriate interventions (Glascoe, 2005; Maraz et al., 2015). Therefore, the thresholds identified in the present study should be regarded as empirical screening values rather than clinically validated diagnostic cut-offs. When used cautiously and interpreted within a broader assessment framework, brief screening instruments may nevertheless contribute to the identification of individuals who warrant further evaluation. In particular, the use of a single, short measure such as the PIUQ-9 may facilitate large-scale screening and help identify potential risk profiles not only for problematic internet use but also for related behaviors such as gaming and gambling. Combined with clinical judgment and subsequent targeted assessment using behavior-specific instruments, this approach may facilitate the identification of individuals who could benefit from further evaluation and preventive support (Vondráčková and Gabrhelík, 2016).

### 4.1 Limitations

This study has some limitations that should be considered when interpreting the results. First, we used a cross-sectional, self-report, survey methodology. Regarding this issue, it should be considered that cross-sectional studies are recognized as robust designs for assessing psychometric properties of measurement tools (Umemneku Chikere et al., 2019), including the estimation of the screening/diagnostic accuracy (sensitivity, specificity and predictive values). And studies where the external reference measures are not strong gold standards (self-reports are an imperfect reference standard), are also common for psychometric purposes (Naaktgeboren et al., 2016).

Second, the low prevalence of the presence of OGD and IGD has impacted on the analysis of the validity of the PIUQ-9 for screening the presence of these two specific online behavioral addictions (particularly in the case of IGD). Nevertheless, it is important to consider that this research was conducted among a non-clinical sample, which, despite being considered high-risk, displays relatively low prevalence rates of these clinical conditions.

On the other hand, the empirically derived screening thresholds identified in this study should be interpreted with caution. Because the PIUQ-9 was developed to assess problematic internet use rather than specific online behavioral addictions, its discriminatory capacity for OGD- or IGD-related difficulties should not be interpreted as equivalent to that of disorder-specific instruments. Consistent with this, the relatively modest specificity values and the corresponding FAR and FDR indicate that the proposed thresholds should be regarded as exploratory screening indicators rather than definitive diagnostic cut-offs. Importantly, these thresholds were derived using established self-report questionnaires as reference measures. Although these instruments provide reasonable operational criteria for identifying individuals at increased risk, they do not constitute independent clinical standards, and the reliance on self-reported measures may introduce shared method variance and influence the observed classification accuracy. Therefore, the clinical validity and generalizability of these thresholds remain to be established. Future research should examine the stability, accuracy, and external validity of these thresholds using independent criteria, including clinician-administered diagnostic assessments, evidence of functional impairment, and, where feasible, objective behavioral indicators. Larger and more heterogeneous samples would also be valuable for evaluating whether the proposed thresholds remain stable across different populations and settings. A related limitation is the lack of previous research establishing comparable screening thresholds for PIU (as measured with the RASNI), OGD, and IGD. Consequently, it was not possible to compare the empirically derived values obtained in the present study with thresholds established in other samples or studies. Future research should address this gap by developing and validating comparable thresholds across populations and instruments, thereby facilitating more robust cross-study comparisons.

Finally, the characteristics of the sample may limit the generalizability of the findings to the broader university student population. Participants were recruited through convenience sampling from a single university and consisted exclusively of first-year psychology students, with women representing a relatively large proportion of the sample. These characteristics may be associated with specific patterns of internet use, awareness and reporting of psychological symptoms, and gaming- or gambling-related difficulties. Therefore, the factor structure, psychometric properties, and empirically derived screening thresholds identified in this study should be interpreted within the characteristics of the present sample and should not be assumed to generalize to students from other academic disciplines, years of study, institutions, geographical regions, or sociodemographic and cultural backgrounds. Future studies should replicate these findings in larger and more heterogeneous multisite samples to examine their stability and external validity across different student populations and settings.

## 5 Conclusion

Young adulthood is a period marked by heightened behavioral changes (Anderson et al., 2017; Arnett, 2000; Sánchez-Fernández and Borda-Mas, 2023). Individuals in this age group commonly engage in extensive internet-based activities for academic, work-related, and leisure purposes, which may increase vulnerability to PIU and related difficulties (Sánchez-Fernández and Borda-Mas, 2023; Shulman et al., 2016). Given the increasing prevalence and potential negative consequences of PIU among university students, brief screening tools with adequate psychometric properties may be useful in this population. In the present study, the PIUQ-9 provided evidence of reliability and validity in a sample of first-year psychology students from a Spanish university. Overall, these findings support the potential utility of the PIUQ-9 as a brief first-stage instrument for identifying students at increased risk of PIU who may benefit from more comprehensive assessment, while its potential use for identifying related behavioral difficulties should be considered exploratory.

## Data Availability

The datasets analyzed during the study are not publicly available due to patient confidentiality and other ethical reasons but are available from the corresponding author on reasonable request. Requests to access the datasets should be directed to sjimenez@bellvitgehospital.cat.
