# Supplementary material for: Early detection of problematic internet use among university students: psychometric validation of the PIUQ-9
Source: Front Psychol. 2026 Sep 30;17:1936216. doi: 10.3389/fpsyg.2026.1936216 (PMC13661367; doi:10.3389/fpsyg.2026.1936216)
Supplement: Supplementary file 1 [file Supplementary_file_1.docx]

**Supplementary Material**

***Table S1 (supplementary)*** *Description of the instruments of the study*

|  | *Number* | Consistency | Total (273) | | Women (180) | | Men (93) | |
| --- | --- | --- | --- | --- | --- | --- | --- | --- |
|  | *Items* | α | Mean | SD | Mean | SD | Mean | SD |
| RASNI Addiction symptoms | 9 | 0.747 | 21.75 | 4.19 | 21.50 | 4.02 | 22.23 | 4.47 |
| RASNI Social use | 8 | 0.764 | 17.57 | 3.71 | 18.20 | 3.61 | 16.35 | 3.60 |
| RASNI Freaky traits | 6 | 0.761 | 9.21 | 2.68 | 8.24 | 2.09 | 11.08 | 2.71 |
| RASNI Nomophobia | 6 | 0.732 | 14.48 | 3.18 | 14.15 | 2.89 | 15.12 | 3.61 |
| RASNI Total | 29 | 0.842 | 63.01 | 9.63 | 62.09 | 8.49 | 64.77 | 11.36 |
| Gaming disorder: number criteria | 9 | 0.720 | 0.81 | 1.32 | 0.37 | 0.96 | 1.68 | 1.48 |
| Gambling disorder: number criteria | 9 | 0.752 | 0.23 | 0.88 | 0.11 | 0.69 | 0.48 | 1.13 |
| UPPS-P Lack premeditation | 11 | 0.898 | 22.77 | 6.72 | 21.12 | 5.03 | 25.98 | 8.29 |
| UPPS-P Lack perseveration | 10 | 0.873 | 21.84 | 6.37 | 20.61 | 5.12 | 24.23 | 7.76 |
| UPPS-P Sensation seeking | 12 | 0.867 | 29.11 | 7.89 | 28.32 | 7.96 | 30.65 | 7.56 |
| UPPS-P Positive urgency | 14 | 0.924 | 24.58 | 8.14 | 23.00 | 7.42 | 27.65 | 8.63 |
| UPPS-P Negative urgency | 12 | 0.872 | 27.89 | 7.42 | 27.27 | 6.89 | 29.09 | 8.25 |
| UPPS-P Total | 59 | 0.946 | 126.20 | 26.74 | 120.32 | 21.54 | 137.58 | 31.81 |
| DERS Non acceptance emotions | 6 | 0.877 | 14.50 | 5.64 | 14.52 | 5.75 | 14.46 | 5.44 |
| DERS Diff. directed behaviours | 5 | 0.859 | 16.23 | 4.38 | 16.04 | 4.27 | 16.60 | 4.60 |
| DERS Impulse control difficulties | 6 | 0.882 | 14.25 | 5.52 | 13.76 | 5.15 | 15.18 | 6.09 |
| DERS Lack emotional awareness | 6 | 0.855 | 14.80 | 4.99 | 14.34 | 5.00 | 15.68 | 4.89 |
| DERS Limited access emotions | 8 | 0.884 | 20.32 | 6.97 | 19.77 | 6.94 | 21.39 | 6.93 |
| DERS Lack emotional clarity | 5 | 0.887 | 12.15 | 4.37 | 12.35 | 4.24 | 11.77 | 4.61 |
| DERS Total | 36 | 0.938 | 92.25 | 22.77 | 90.79 | 22.19 | 95.09 | 23.71 |
| SCL-90R Somatization | 12 | 0.860 | 1.15 | 0.71 | 1.26 | 0.73 | 0.94 | 0.60 |
| SCL-90R Obsessive/compulsive | 10 | 0.879 | 1.39 | 0.88 | 1.61 | 0.81 | 0.96 | 0.85 |
| SCL-90R Interpersonal sensitivity | 9 | 0.864 | 1.47 | 0.88 | 1.65 | 0.91 | 1.12 | 0.69 |
| SCL-90R Depressive | 13 | 0.896 | 1.28 | 0.82 | 1.47 | 0.80 | 0.92 | 0.75 |
| SCL-90R Anxiety | 10 | 0.841 | 1.09 | 0.69 | 1.21 | 0.73 | 0.86 | 0.54 |
| SCL-90R Hostility | 6 | 0.816 | 0.97 | 0.78 | 0.93 | 0.76 | 1.06 | 0.80 |
| SCL-90R Phobic anxiety | 7 | 0.775 | 0.74 | 0.67 | 0.85 | 0.70 | 0.55 | 0.56 |
| SCL-90R Paranoid Ideation | 6 | 0.741 | 1.24 | 0.78 | 1.31 | 0.81 | 1.09 | 0.71 |
| SCL-90R Psychotic | 10 | 0.854 | 0.76 | 0.72 | 0.89 | 0.71 | 0.52 | 0.68 |
| SCL-90R GSI | 90 | 0.975 | 1.14 | 0.64 | 1.27 | 0.65 | 0.88 | 0.55 |
| SCL-90R PST | 90 | 0.975 | 52.02 | 21.31 | 56.57 | 20.11 | 43.22 | 20.90 |
| SCL-90R PSDI | 90 | 0.975 | 1.87 | 0.47 | 1.93 | 0.48 | 1.76 | 0.43 |

*Note.* α: Cronbach’s alpha in the study. SD: standard deviation.

RASNI: Scale of Risk of Addiction to Social Networks and Internet (ERA-RSI in Spanish version).

UPPS-P: Impulsive Behavior Scale. DERS: Difficulties in Emotion Regulation Scale

SCL-90R: Symptom Checklist-90-Revised.

***Table S2 (supplementary)*** *Conversion table for PIUQ-9 raw scores into percentile ranks and standardized T-scores*

| **Raw** | **General** | | **Obsession** | | **Neglect** | | **Control** | | **Neglect/Control** | | **Raw** |
| --- | --- | --- | --- | --- | --- | --- | --- | --- | --- | --- | --- |
| **score** | **Percent.** | **T** | **Percent.** | **T** | **Percent.** | **T** | **Percent.** | **T** | **Percent.** | **T** | **score** |
| **0** | 1 | 25 | 7 | 35 | 1 | 28 | 2 | 29 | 1 | 25 | **0** |
| **1** | 1 | 27 | 13 | 39 | 4 | 32 | 4 | 33 | 1 | 27 | **1** |
| **2** | 2 | 29 | 22 | 42 | 9 | 37 | 9 | 37 | 2 | 30 | **2** |
| **3** | 2 | 30 | 34 | 46 | 18 | 41 | 18 | 41 | 4 | 32 | **3** |
| **4** | 4 | 32 | 48 | 49 | 32 | 45 | 31 | 45 | 6 | 35 | **4** |
| **5** | 5 | 34 | 62 | 53 | 50 | 50 | 46 | 49 | 10 | 37 | **5** |
| **6** | 7 | 36 | 74 | 57 | 67 | 54 | 62 | 53 | 15 | 40 | **6** |
| **7** | 10 | 37 | 84 | 60 | 81 | 59 | 76 | 57 | 21 | 42 | **7** |
| **8** | 13 | 39 | 91 | 64 | 91 | 63 | 87 | 61 | 29 | 44 | **8** |
| **9** | 18 | 41 | 96 | 67 | 96 | 68 | 94 | 65 | 38 | 47 | **9** |
| **10** | 22 | 42 | 98 | 71 | 99 | 72 | 97 | 69 | 48 | 49 | **10** |
| **11** | 28 | 44 | 99 | 74 | 100 | 77 | 99 | 73 | 57 | 52 | **11** |
| **12** | 34 | 46 | 100 | 78 | 100 | 81 | 100 | 77 | 66 | 54 | **12** |
| **13** | 40 | 48 |  |  |  |  |  |  | 75 | 57 | **13** |
| **14** | 47 | 49 |  |  |  |  |  |  | 82 | 59 | **14** |
| **15** | 54 | 51 |  |  |  |  |  |  | 88 | 62 | **15** |
| **16** | 61 | 53 |  |  |  |  |  |  | 92 | 64 | **16** |
| **17** | 67 | 54 |  |  |  |  |  |  | 95 | 66 | **17** |
| **18** | 73 | 56 |  |  |  |  |  |  | 97 | 69 | **18** |
| **19** | 78 | 58 |  |  |  |  |  |  | 98 | 71 | **19** |
| **20** | 83 | 60 |  |  |  |  |  |  | 99 | 74 | **20** |
| **21** | 87 | 61 |  |  |  |  |  |  | 100 | 76 | **21** |
| **22** | 90 | 63 |  |  |  |  |  |  | 100 | 79 | **22** |
| **23** | 93 | 65 |  |  |  |  |  |  | 100 | 81 | **23** |
| **24** | 95 | 66 |  |  |  |  |  |  | 100 | 84 | **24** |
| **25** | 97 | 68 |  |  |  |  |  |  |  |  | **25** |
| **26** | 98 | 70 |  |  |  |  |  |  |  |  | **26** |
| **27** | 98 | 72 |  |  |  |  |  |  |  |  | **27** |
| **28** | 99 | 73 |  |  |  |  |  |  |  |  | **28** |
| **29** | 99 | 75 |  |  |  |  |  |  |  |  | **29** |
| **30** | 100 | 77 |  |  |  |  |  |  |  |  | **30** |
| **31** | 100 | 78 |  |  |  |  |  |  |  |  | **31** |
| **32** | 100 | 80 |  |  |  |  |  |  |  |  | **32** |
| **33** | 100 | 82 |  |  |  |  |  |  |  |  | **33** |
| **34** | 100 | 84 |  |  |  |  |  |  |  |  | **34** |
| **35** | 100 | 85 |  |  |  |  |  |  |  |  | **35** |
| **36** | 100 | 87 |  |  |  |  |  |  |  |  | **36** |
|  | *Mean* | *SD* | *Mean* | *SD* | *Mean* | *SD* | *Mean* | *SD* | *Mean* | *SD* |  |
|  | 14.42 | 5.82 | 4.17 | 2.81 | 5.02 | 2.24 | 5.24 | 2.46 | 10.26 | 4.09 |  |

*Note*. Percent.: percentile. T: standardardized T score. Sample size: *N* = 273.

***Figure S1 (supplementary)*** *Selection of the optimal cut-off for the PIUQ-9 one general factor to identify OGD and IGD: ROC analysis*

*Note*. Se: sensitivity (%). Sp: specificity (%). FDR: false discovery rate (%).

The dashed vertical line indicates the optimal cutoff point. Sample size: *N* = 273.
